# Clinical Guide to Obsessive Compulsive and Related Disorders

**DOI:** 10.1192/pb.bp.114.049510

**Published:** 2015-06

**Authors:** Ijeoma E. Onuba

This book gives an overview of obsessive–compulsive disorder (OCD), hoarding disorder, body dysmorphic disorder, excoriation (skin picking) and trichotillomania, all listed in DSM-5 under ‘obsessive–compulsive and related disorders’. The authors have also included hypochondriasis and tic disorder as some of the related disorders.

The book is divided into three parts. The first part gives a general overview and evaluation of the disorders. The second part discusses each disorder in detail, focusing on the clinical description, diagnosis, comorbidity, course and prognosis, differential diagnosis and treatment. Scales that can be used in monitoring treatment are included in the appendix at the end of the book. The final part is titled ‘special clinical considerations’ and addresses areas such as treatment resistance, treatment of children and people with intellectual disability, and alternative treatments. This part also mentions neurosurgery for OCD and the ethical dilemmas associated with this approach. The appendices have a list of suggested further reading and contact details of organisations and treatment centres.

A useful resource for trainees and students is a table in the first chapter, which shows types of obsessions and compulsions with good examples. I also like the way the authors describe how to differentiate the symptoms associated with each disorder and normal behaviour. The book also gives practical advice on how to screen for these disorders. The response rates to treatments are discussed and some chapters also mention research work.

There is a table summarising pharmacological treatment for each disorder when managing children and I wished a similar table was done at the end of part two, which could be used as a quick reference guide. The book’s title may mislead readers who are looking for information on hypochondriasis and tic disorder.

I would recommend this book for health professionals, students and even patients and their carers. It is well written, concise and easy to follow.

